# Early oleate deficiency leads to severe defects in fetal rat liver development

**DOI:** 10.22038/ijbms.2019.35084.8345

**Published:** 2019-09

**Authors:** Fatemeh Mohammadzadeh, Alireza Alihemmati, Abbas Pirpour Tazehkand, Masoud Darabi, Amir Mehdizadeh

**Affiliations:** 1 Liver and Gastrointestinal Diseases Research Center, Tabriz University of Medical Sciences, Tabriz, Iran; 2 Department of Anatomical Sciences, Faculty of Medicine, Tabriz University of Medical Sciences, Tabriz, Iran; 3 Department of Biochemistry and Clinical Laboratories, Faculty of Medicine, Tabriz University of Medical Sciences, Tabriz, Iran; 4 Endocrine Research Center, Tabriz University of Medical Sciences, Tabriz, Iran; 5 Comprehensive Health Lab, Tabriz University of Medical Sciences, Tabriz, Iran

**Keywords:** Development, Embryo, Hepatocytes Monounsaturated fatty acids, Pregnancy

## Abstract

**Objective(s):**

Oleate can be produced through de novo synthesis, which contributes to biological processes and signaling pathways. However, the role of this non-essential fatty acid in hepatic development remains unclear. The current study aimed to evaluate the influence of early oleate deficiency induced by the inhibitor of de novo oleate synthesis MF-438 on fetal rat liver development.

**Materials and Methods:**

Female Wistar rats with an average weight of 200±20 g were subjected to this study. After mating, pregnant rats were divided into three groups and gavaged with the vehicle, MF 438 or MF-438 plus oleate from day 3 of pregnancy for five days. Obtained fetuses were sacrificed and the liver tissues were retrieved. Hepatic morphological index, biochemical markers, and gene expression of hepatic development markers were analyzed using Hematoxylin-Eosine, spectrometry, and real-time PCR techniques, respectively.

**Results:**

Relatively, deficient morphological indices and hepatic maturation markers were observed in fetus livers of the inhibitor-treated group. In comparison to the other two groups, total hepatic protein and glycogen content were increased with treatment of MF-438 plus oleate. Hepatocyte nuclear factor 1α, alpha fetoprotein, albumin, and cytochrome P450 gene expression were also significantly increased in the group treated with both MF-438 and oleate.

**Conclusion:**

Our data indicate that oleate availability during early embryo development is linked with fetal rat liver development.

## Introduction

Embryo hepatic development is a multi-stage process that may be affected by maternal and embryo metabolism status (1). Any defect in this process will result in clinical features relevant to deficient hepatic growth at or after birth. For instance, newborn jaundice is a common clinical complication related to the disability of hepatic cells to detoxify metabolic end-products (2). However, no certain pathogenesis is explained for some hepatic insufficiency, including jaundice at birth. Therefore, study of the potential factors implicated in embryo hepatic evolution seems to be critical. Recent studies have considered the oleate-rich diet as a beneficial regimen for many biological pathways, including hepatic gene expression and energy homeostasis (3, 4). Oleate rich diet can alleviate hypercholesterolemia and LXR-dependent hepatic lipogenesis in rats (3). Accordingly, oleate can improve hepatic function in non-alcoholic fatty liver disease and non-alcoholic steatohepatitis models (5). However, the role of oleate in liver development remains unknown. Especially, no study has yet examined the influence of oleate availability on fetal hepatic development.

**Figure 1:**
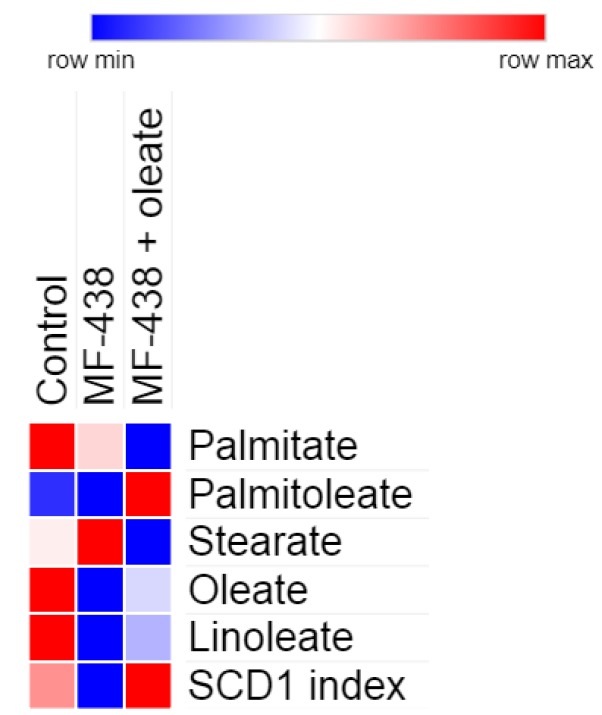
Heatmap comparison of changes in hair follicle fatty acids and stearoyl-CoA desaturase (SCD1) index of the studied pregnant rats on gestation day 12. The analyzed data are normalized to the amount of internal standard

**Figure 2:**
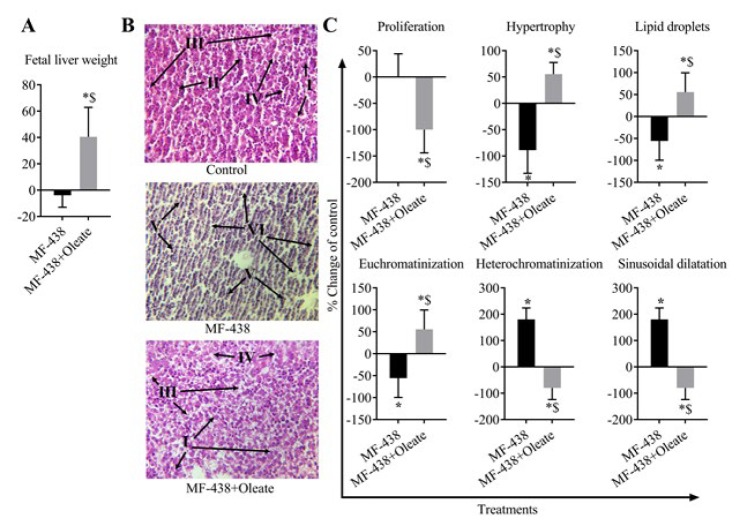
Hepatic fetus morphological changes in the studied groups. (A) Fetus liver weights in experimental groups. Data are presented as percent change from controls. (B) Sectional micrograph of rat hepatic fetal tissue. In the hepatic cells control group, euchromatinization and normal sinusoids are obvious. Some cells also exhibit heterochromatinization with accumulated cytoplasmic lipid droplets. Sectional micrograph of hepatic tissue after SCD1 inhibition illustrated atrophic cells with heterochromatinization and dense nuclei. Neither sinusoidal dilatation nor cellular lipid accumulation was observed. Sectional micrograph of hepatic tissue after combined MF-438 and oleate supplementation showed cells with normal sinusoids, hypertrophy, and euchromatinization associated with accumulated lipid droplets. Euchromatinization, heterochromatinization, lipid droplets, normal sinusoids, autotrophic cells, and sinusoidal dilatation are noted by I-VI arrows, respectively. (C) Semi-quantitative representation of morphological changes in experimental groups. Intensity of changes is reported following examination of 5 microscopic fields for each index. Data are presented as percent change from controls (*P-value<0.001 vs control, $*P*-value<0.001 vs MF-438, n=6 for each group)

**Figure 3:**
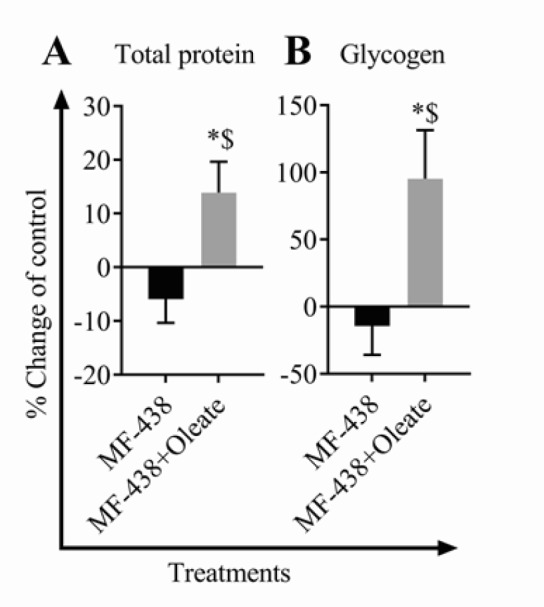
Total protein (A) and glycogen (B) content of fetus liver in the experimental groups (*P-value<0.001 vs control, $*P*-value<0.001 vs MF-438, n=6 for each group)

**Figure 4:**
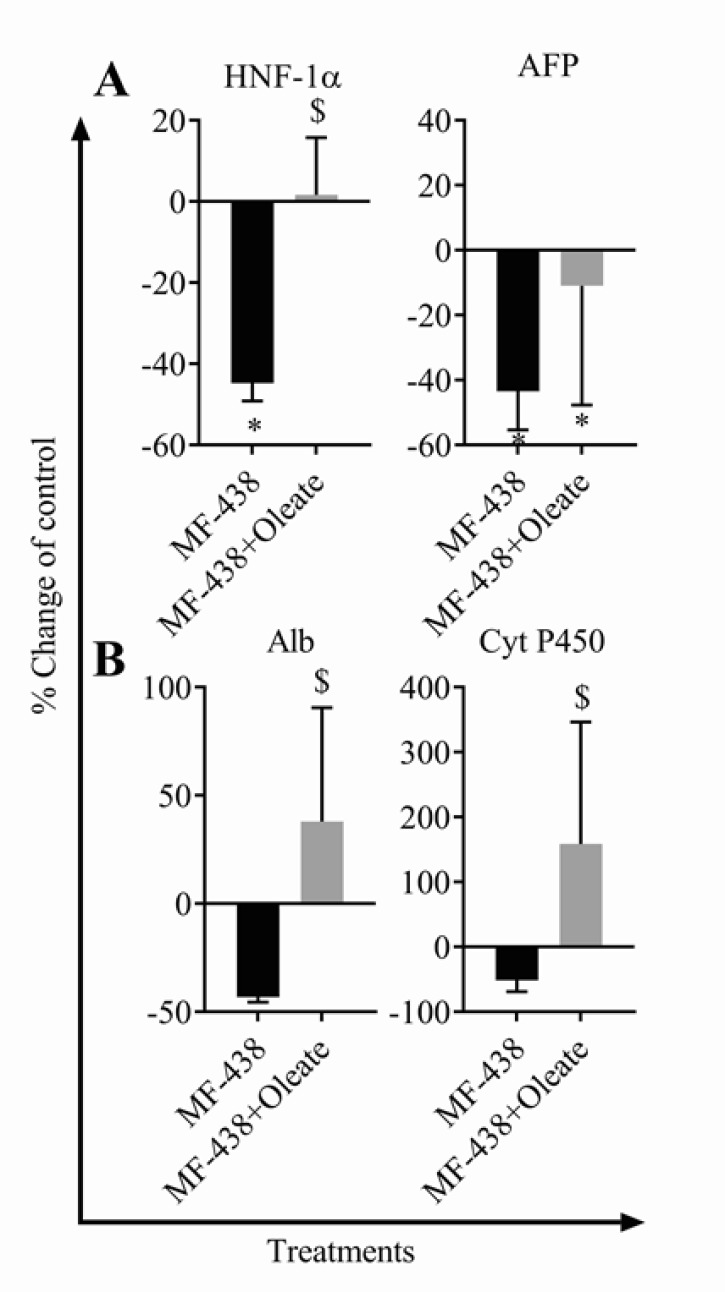
The effects of stearoyl-CoA desaturase 1 (SCD1) inhibition and oleate supplementation on gene expression of hepatic development markers. HNF 1α; hepatocyte nuclear factor-1α, AFP; alpha-fetoprotein, Alb; albumin, Cyt P450; cytochrome P450 (*P-value<0.001 vs control, $*P*-value<0.001 vs MF-438, n=6 for each group)

Stearoyl-CoA desaturase 1 (SCD1) is a key enzyme in *de novo* synthesis of lipids through desaturation of fatty acids. This enzyme is highly expressed in lipogenic tissues such as liver where it actively produces precursors of cellular components and active metabolites (6). Additionally, cellular ratio of unsaturated to saturated fatty acids is thoroughly correlated with cell signaling, growth, differentiation regulation, membrane fluidity, and *in vitro* embryo development (7, 8). In our previous study, chemical inhibition of SCD1 during primary commitment phase of pluripotent stem cells differentiation to hepatocytes led to a decrease in hepatic markers production including hepatocyte nuclear factor (HNF)-4α and alpha-fetoprotein (AFP) *in vitro* in the obtained hepatocyte-like cells, which was effectively reversed by adding exogenous oleate (9). HNF-1α and HNF-4α regulate the transcription of essential genes for hepatic lineage reprogramming (10). Early expression of AFP before liver morphogenesis indicates the role of this protein as a signaling molecule in endodermal cells for their commitment toward hepatic lineage (11). In a study on a group of signaling proteins, SCD1 products were found to be critical for producing active, lipid-modified Wnt proteins (12). Furthermore, SCD1 expression level was related to adipocyte differentiation in the mouse embryonic fibroblast cell line 3T3-L cells (13). In another study, expression levels of SCD1 and fatty acid synthase were also found to be necessary for hepatic metabolic function and maturation (14). Formation of fetal rat liver is initiated from the anterior foregut endoderm at day 10 of pregnancy. Appearance of hepatic diverticulum on gestation day 12 is associated with hepatoblast formation. The liver tissue evolution is continued until day 15 ^1^. However, earlier stages of embryo development may share a higher degree of importance for the next liver development, growth, and function. The present study aimed to evaluate the influence of early oleate deficiency on the late fetal rat liver development.

## Materials and Methods

***Animals and treatments***

This experimental study was performed on female Wistar rats under controlled conditions. The average weight of rats was 200±20 g. All animals were housed under a 12 hr light (7 am-7 pm)/dark cycle and were fed a standard laboratory diet and water *ad libitum*. The protocol of this study was approved by the local ethics committee at Tabriz University of Medical Sciences (code: IR.TBZMED.REC.1395.701). After two weeks of adaptation, female rats with a positive estrous cycle test were mated (15). Briefly, vaginal discharge smears were prepared following injection of 200 µl normal saline into the rat vagina and evaluated for the presence of nucleus-free corn-shaped epithelial cells. The selected female rats were then mated with the male counterparts for 24 hr; the first day after mating was considered as day 0 of pregnancy. Then pregnant rats were divided into three groups and gavaged with the vehicle, 5 mg/kg/day of the SCD1 inhibitor MF-438 (Focus Biomolecules, USA, 921605-87-0) or MF-438 plus 300 mg/kg/day oleate (Carlo Erba, France, 305704). One of the major objectives of this study was to investigate the mechanism related to the adverse effect of SCD1 inhibition on fetus hepatic maturation. It is notable that the products of SCD1 are not limited to oleate; palmitoleate is another direct product of SCD1. Therefore, we included the experimental group receiving a combination of SCD1 inhibitor and oleate. The oral inhibitor solution was prepared by mixing 6 mg of MF-438 with 1200 µl of starch solution (27% in distilled water). The prepared solution was gavaged to the rats in days 3–7 of pregnancy for five days. For MF-438 and oleate combination, 6 mg of MF-438 and 400 µl of pure oleate were mixed with 1200 µl of starch solution and were gavaged. Control rats received only starch solution in days 3–7 of pregnancy. Pregnant rats were anesthetized using 100 mg/kg ketamine combined with 10-20 mg/kg xylene, and sacrificed by guillotine on day 19 of pregnancy. Obtained fetuses were also sacrificed through spinal cord interruption and liver tissues were retrieved. 

***Fatty acid composition analysis***

SCD1 is highly expressed in skin and hair follicles (16, 17). To evaluate the effectiveness of SCD1 inhibitor, fatty acid composition of hair follicles, as a non-invasive sampling method in pregnant rats (3–5 follicles), was analyzed on gestation day 12 using a gas-liquid chromatography instrument (Buck Scientific 610, USA). Briefly, samples were transferred to glass tubes. Then, 2 ml of methanol/hexane (4:1) (Merck, 106009/109687, Darmstadt, Germany) and 0.2 ml of acetyl chloride (Merck, 822252, Darmstadt, Germany) were added, and the tightly capped tubes were placed in boiling water for 1 hr. Afterward, the tubes were cooled down and 5 ml of 6% K_2_CO_3_ was added to the tubes, and the hexane supernatant phase was recovered and subjected to gas-liquid chromatography analysis. 

***Histology***

The wet weights of fetus livers were measured using a sensitive analytical balance. For histopathological studies, fetal liver tissue was isolated and placed in 10% formalin solution. The samples were dehydrated in ethanol and cleared by xylene and embedded in paraffin. Several serial sections (5 μm thicknesses) were prepared after tissue processing steps and stained by Hematoxylin-Eosin (Sigma Aldrich, Germany, MHS16) for microscopic observations. The stained sections were evaluated by a blind histologist for hepatocyte proliferation, hypertrophy, lipid accumulation, euchromatinization, heterochromatinization, and sinusoidal dilatation.

***Analysis of fetus hepatic glycogen content***

Fetal hepatic glycogen content was analyzed according to the Zhang protocol (18). Briefly, 0.5 ml of 2 M HCl was prepared in a 2 ml microtube for each sample as the control group. For measuring free glucose, 2 M HCl was replaced with 2 M NaOH. The tubes were placed in boiling water for 3–5 min, centrifuged briefly and weighed after wiping using an analytical balance. An amount of 10 to 20 mg of frozen liver tissue was added to the hot tubes containing HCl or NaOH, and the weight of tubes was measured again. Afterward, tubes were tightly sealed and placed in boiling water for 1 hr. In order to achieve complete hydrolysis, tissues were minced by a scissor for 5 min and were shaken every 10 min. The tubes were cooled down in room temperature and centrifuged briefly. The original weight of tubes was also reconstituted by adding double distilled water (ddH_2_O). Finally, the reaction medium was neutralized by adding 0.5 ml of 2 M HCl or NaOH, accordingly and centrifuged at 3000 rpm for 10 min. The glucose concentration of obtained supernatants was evaluated using Pars Azmoon kit (Iran, Tehran, 117500).

***Evaluation of fetus hepatic total protein***

Fetal hepatic total protein was evaluated according to Lowry’s method (19). Briefly, the Lowry solution was prepared by adding 9.7, 0.15, and 0.15 ml of reagent A (3% Na_2_CO_3_ in 0.15 M NaOH), reagent B (2% potassium sodium tartrate in ddH_2_O), and reagent C (1% CuSO_4_.5H_2_O in ddH_2_O), respectively. Then, 100 mg of frozen liver tissue was homogenized in 0.9% NaCl and centrifuged at 3000 rpm for 5 min and the supernatant was subjected to total protein evaluation. Volumes of 5 and 395 µl of supernatant and ddH_2_O were added to 1 ml of the Lowry solution. Bovine serum albumin (Alb) was used as the standard. Tubes were incubated in room temperature for 10 min. Next, 100 µl of Folin solution (1:4 diluted by ddH_2_O) was added to all tubes and incubated for an additional 30 min in room temperature. Finally, the optical density of all tubes was measured in 750 nm wavelength. The test tube with 1 ml distilled water served as the blank. 

***Quantitative real-time PCR***

Total RNA of liver tissue was extracted using a QIAGEN Rneasy Mini kit (USA, Valencia, 74104). Quality and quantity of the extracted RNA were evaluated by agarose gel electrophoresis and nanodrop instrument (ND-1000, Thermo Scientific, Wilmington, DE, USA), respectively. About 0.5–3 µg of total RNA was transcribed reversely into c-DNA using a Takara Primescript RT reagent kit (Cat No RR037Q, Tokyo, Japan). SYBR green (Cat. No. RR820Q, Takara, Tokyo, Japan) master mix was used for qRT-PCR using a Mic q-PCR system (Bio molecular systems, Sydney, Australia). Glyceraldehyde-3-phosphate dehydrogenase (GAPDH) mRNA was measured as the internal standard. Primers were analyzed using NCBI primer BLAST and Oligo7 software (Molecular Biology Insights, Inc.). The optimum Tm for rat AFP, Alb, HNF1α, Cyt P450, and GAPDH was 59 ^°^C. Primer sequences are listed in supplementary Table 1.

***Statistical analysis***

Data from 21 fetal liver tissues obtained from 3 pregnant rats in each group were analyzed using GraphPad Prism ANOVA followed by *post hoc* Tukey’s test. To simplify data presentation, all results were expressed as % change from control, and all quantitative data were presented in supplementary data. Heatmap was drawn by Morpheus (Broad Institute, USA). A *P*-value of <0.05 was considered as statistically significant. 

## Results

***MF-438 led to significant reduction in tissue oleate of pregnant rats.***

Analysis of the fatty acid composition of hair follicles in the pregnant rats indicated that inhibitor administration during early pregnancy led to a significant decrease in oleate and SCD1 activity index, lasting several days after treatment (Figure 1).

***Oleate deficiency led to severe defects in fetal liver morphologic indices***

Morphologic changes of fetus livers were evaluated by histochemical staining. Higher heterochromatinization and hepatic sinusoidal dilatation was observed in SCD1 inhibitor treated group, compared to control (both +180%, *P-*value<0.001) and MF-438 plus oleate (both +260%, *P-*value<0.001) groups. Hypertrophy, accumulation of lipid droplets and euchromatinization indices were also lower in SCD1 inhibitor treated group compared to controls (<-55%, *P-*value<0.001) and MF-438 plus oleate (<110%, *P-*value<0.001) groups. As shown in Figure 2, a higher liver weight was observed in MF-438 plus oleate treatment compared to control and MF-438 groups (+40.42%, *P-*value=0.001 and +44.29%, *P-*value<0.001, respectively). Additionally, higher hepatic proliferation, hypertrophy, accumulation of lipid droplets, and euchromatinization were observed in MF-438 plus oleate treatment.

***Combined MF-438 and oleate treatment during the early pregnancy increased fetus liver total protein and glycogen content.***

MF-438 treatment during gestation days 3–7 decreased liver total protein and glycogen content. However, the total protein content of fetus liver was increased in MF-438 plus oleate treated group compared to both control and MF-438 only treated groups (+13.89% and +19.83%, *P-*value<0.001, respectively; (Figure 3A). Similar results in fetus glycogen content were seen after MF-438 plus oleate treatment (+95.3% and +109.73%, *P-*value<0.001, respectively) (Figure 3B).

***Combined MF-438 and oleate treatment increase gene expression of maturation markers in fetus liver.***

As shown in Figure 4, inhibition of SCD1 resulted in a significant reduction in HNF1α and AFP expression (-44.8% and -43.49%, *P-*value<0.001). However, combined MF-438 plus oleate at the early pregnancy elevated expression of hepatic markers compared to MF-438 group, indicating the compensatory effects of exogenous oleate on SCD1 inhibition (+46.4% , +54.43%, , +81.6%, and +210.15%, *P-*value<0.001 for HNF1α, AFP, Alb, and Cyt P450, respectively). 

## Discussion

Even though oleate is both nutritionally and metabolically important, no study has yet examined its association with embryo development and organogenesis. In the present study, the effects of oleate deficiency on fetal rat liver development were evaluated in pregnant rats. To induce oleate deficiency, MF-438 was used as a specific SCD1 inhibitor, and to recover this effect, oral oleate was administered. It has been previously reported that non-toxic dose of SCD1 inhibitor during endodermal induction *in vitro* postponed the production of AFP (9). Later in induced hepatic differentiation, overall levels of Alb and urea were also decreased at hepatic maturation stage, which were recovered by adding oleate (9). The findings of the present study indicated apparent histopathological markers of fetal hepatic insufficiency due to the induction of oleate deficiency. As expected, exogenous administration of oleate as the major product of SCD1 improved growth markers. Similar effects were also observed in expression of hepatic function markers. Both palmitoleate and oleate are the products of SCD1. Our findings, however, confirm that supplementation of exogenous oleate alone can compensate SCD1 role in fetal liver development.

The mechanism related to the beneficial effect of oleate on liver development is yet to be examined. However some studies in non-liver tissues have shown similar effects of oleate on differentiation. For example, Lee *et al*. (20) reported that myoblasts treated with oleate exhibit higher morphological differentiation markers compared to controls. Coupled with myogenic differentiation similar effects were observed in mitochondrial biogenic genes, including peroxisome proliferator-activated receptor-γ coactivator 1-alpha, nuclear respiratory factor, and mitochondrial transcription factor A. Stem cell differentiation is accompanied with mitochondrial biogenesis (21, 22). In embryonic stem cells, mitochondrial phenotype is described as “immature” consisting of low numbers of mitochondria, poorly developed crista and a perinuclear location (23). In contrast, differentiated cells show higher mitochondrial mass, mitochondrial DNA (mt-DNA), expanded mitochondrial network, and a shift towards oxidation/phosphorylation to supply their energy demands (24). In this regard, Yuzefovych *et al*. (25) showed that in contrast to palmitate, oleate supplementation improves mitochondrial function by inhibiting reactive mitochondrial oxygen species production and DNA damage in rat skeletal muscle cells. Furthermore, this fatty acid prevents palmitate-induced mt-DNA damage and apoptosis and increases ATP level and cell survival. 

In another study on myoblast differentiation to myotubes (26), supplementation of mono- and poly-unsaturated fatty acids, including oleate, led to a significant caveolin-3 expression. Additionally, oleate supplementation increased its elongated products, including 22:1n-9 and 22:4n-6 in phosphatidylethanolamine and phosphatidylethanolamine/phosphatidylserine ratio in myotubes (26). Caveolin-3 and oleate and its elongated derivatives are all beneficial to membrane fluidity, which is directly related to stem cell differentiation (26). Probably, oleate by a similar mechanism improves the hepatocyte formation in the primary stages of fetal growth through increases in mitochondrial activity and membrane dynamics. 

Accordingly, a recent study analyzed the effects of saturated and unsaturated fatty acids on HepG2 cell plasma membrane protection and fluidity (27). The results illustrated protective effects of unsaturated fatty acids, including oleate, against cytotoxicity, associated with increased membrane fluidity compared to palmitate as a saturated fatty acid (27).

In line with our findings, the beneficial effects of oleate have been shown on germ cell competence to support embryo *in vitro* development (28). In the present study, oleate supplementation in an SCD1 deficient animal model led to improvement of hepatic development markers even compared to the control group. In this regard, it seems that endogenous production of oleate by SCD1 has no advantages against the nutritional supply of this fatty acid. Examination of this hypothesis needs to be confirmed through single supplementation of oleate during fetal development. This study simultaneously evaluated phenotype and molecular hepatic development markers *in vivo. *The study was focused on the effect of oleate deficiency during the early phase of pregnancy. Oleate and SCD1 activity may also be important in other pregnancy periods. At the beginning of pregnancy, however, pluripotent embryonic cells are more dependent on maternal support, and the status of maternal oleate seems to be more important for embryo development. The findings of this study support the idea that end-stage liver development is dependent on sufficient oleate availability during early embryo development.

## Conclusion

The presented data indicates that oleate availability for early rat embryo may be a critical parameter in fetal liver development. Clinical trials on oleate supplementation during early pregnancy can provide a new strategy for effective and safe fetal embryo intervention, potentially applicable for controlling term and preterm neonatal liver insufficiency.
